# Supplementary material for: Vertical versus horizontal Spatial-Numerical Associations (SNA): A processing advantage for the vertical dimension
Source: PLoS One. 2022 Aug 25;17(8):e0262559. doi: 10.1371/journal.pone.0262559 (PMC9409557; doi:10.1371/journal.pone.0262559)
Supplement: Attachment — Submitted filename: ResponsesWithLetter.pdf [file pone.0262559.s001.pdf]

Dear Dr Jérôme Prado,

Thank you for the opportunity to revise our manuscript PONE-D-21-40286 “The Vertical versus Horizontal SNARC Effect: A processing advantage for being upwards” for consideration in PLOS ONE. I apologise that we are slightly delayed in our response due to some ongoing COVID19 disruptions to our work flow management. We have now been able to implement a full revision based on the advice of the two reviewers, and yourself.

Due to the detailed nature of the requested amendments, here in our response/rebuttal letter we have employed a table form of logging responses as I hope this makes it easier for both yourself, and reviewers, to understand how the manuscript has been modified in relation to specific comments. As requested, we provide two versions of the manuscript including with track changes on, and also a clean fully revised version.

Please note that on the advice of reviewer 2 we have revised the manuscript title to “***Vertical versus Horizontal Spatial-Numerical Associations (SNA): A processing advantage for the vertical dimension***”.

As per point 4 in the Table below on funding statements and the instruction “*Please include this amended Role of Funder statement in your cover letter; we will change the online submission form on your behalf.*”; here I inform you we have made this modification as requested in point 4. I believe I have also already declared that I am an AE for PLoS One; but it will be best to make sure this information is correctly logged if anything changes due to the point about funding declarations.

I also need to inform you that, due to some positional changes (and again COVID19 disruptions), some author emails have changed relative to initial submission. Dr Scarlett Howard is now at Monash University (new email scarlett.howard@monash.edu), Dr Eugene Chan is at Toronto Metropolitan University, Ted Rogers School of Management, Toronto, Canada (e34chan@ryerson.ca) and I have just been informed that my RMIT position will likely end 30th June 2022, and so my listed ongoing Monash University (adjunct) position email may be required for communication (adrian.dyer@rmit.edu.au). I have indicated both emails in revised manuscript.

In addition, our respective ORCID numbers are:
Luke Greenacre 0000-0002-6029-6523
Jair E. Garcia 0000-0001-8456-4759
Eugene Chan 0000-0002-6847-6545
Scarlett R. Howard 0000-0002-1895-5409
Adrian G. Dyer 0000-0002-2632-9061.

We are very grateful for the expert advice on how to modify our study, and we look forward to hearing feedback on our revised and improved study.

Yours sincerely, Adrian G. Dyer, on behalf of all authors.

**EDITOR**	
**Comment (THEM)**	**Reply (US)**
As you can see in the two reviews at the bottom of this email, the reviewers disagree on whether the manuscript is suitable for publication in PLOS ONE. While the first reviewer suggests a rejection, the second reviewer is more positive and points out that your manuscript might make an interesting contribution to the literature. Based on these reviews and my own reading of the manuscript, I am willing to give you the opportunity to revise and resubmit the paper. However, I urge you to carefully consider the different points raised by the reviewers.	We wish to thank the editor and reviewers for their thoughts and comments. We have carefully addressed each of the points below having broken down each of the comments into this table and responding to each individually.
Most notably, both experts point out that a shortcoming of your task is that participants were only asked to select the largest (and not the smallest) number. You therefore either need to convincingly explain why this task feature did not affect your results, or (preferably) run an additional experiment in which you present participants with the missing condition.	This is a very important issue raised in the reviews. There are several reasons for why we employed a task where the greater (and not lesser) was selected by participants.<br><br>The reviews implied the ‘lesser’ was a missing condition in the current experiment, however there are theoretical bases to believe it is not a ‘parallel’ to the ‘greater’ as it would invoke different processing in the participants that would have made the greater and lesser not truly comparable. This is because of the presence of the subitizing range in the stimulus.<br><br>In our method we used a full combinatorial of all pairwise single digit numbers (1 thru 9). The list of pairs is thus 36 trials (ignoring direction/screen position which is balanced in the design).<br><br>{1,2} {1,3} {1,4} {1,5} {1,6} {1,7} {1,8} {1,9} {2,3} {2,4} {2,5} {2,6} {2,7} {2,8} {2,9} {3,4} {3,5} {3,6} {3,7} {3,8} {3,9} {4,5} {4,6} {4,7} {4,8} {4,9} {5,6} {5,7} {5,8} {5,9} {6,7} {6,8} {6,9} {7,8} {7,9} {8,9}<br><br>When doing the 'greater', we get the following characteristics for the target/pair: target is 5 or above for 30 pairs (of which 10 have both numbers being 5 or above); target is 4 or below for 6 pairs<br><br>When choosing the 'lesser', we get the following characteristics for the target/pair: target is 5 or above for 10 pairs; target is 4 or below for 26 pairs (of which 6 have both numbers being 4 or below)<br><br>So the two are not exact opposites even though they draw on the same combinatorial due to the subitizing threshold impacting where the

	information is going to arise in the design. The 'lesser' is thus not just another (opposing) condition in the experiment, but will elicit an entirely new set of information that would need to be analysed separately. Of particular note is that it is numbers above the subitizing range where we would expect to see the biggest changes in processing efficiency and accuracy due to size effects. Hence, we sought more observations for that higher range, hence chose the ‘greater’ to give us more instances of the target being 5 or beyond.<br><br>At the time of designing the experiment the research team decided that the ‘greater’ was the core experiment, and the ‘lesser’ a distinct experiment. As the ‘greater’ contained the most (statistical) information for testing our hypothesis. Our ethics application was made and approved to collect that data.<br><br>We also cannot collect this ‘lesser’ data by itself after the fact. We would need to randomly allocate participants between the ‘greater’ and ‘lesser’ experiments to prevent sample selection bias. Further, the pandemic has happened since the ‘greater’ data was collected so there is a case for changes in how the sample may behave from then to now. We would need to discard the existing data and recollect both the lesser and greater at the same time. Our ethics would not allow us to disregard the original greater data.<br>We would argue that the request for the ‘lesser’ data is not practical, and indeed the data in our research is entirely sound – more data on the ‘lesser’ will not make our present data more sound.
Another important issue raised by reviewer #1 concerns the heterogeneity in native languages of the participants. You also need to carefully address this point, possibly with additional analyses.	We have revised the analysis here considerably. We have now provided a larger array of demographic information in addition to language orientation to show the potential bases for heterogeneity.<br>The native language component of the analysis has now been more clearly framed as testing a post-hoc moderator.<br>We have also expanded the analysis presented, particularly for the reaction time data, as the previous presentation of data led to some confusion in the interpretation. We now present both groups (LTR and non-LTR groups) and explain how the results reinforce the core findings of the dominance of the vertical orientation in SNARC.
Finally, both reviewers suggest changes in the structure of the manuscript, which I strongly encourage you to take into	We have revised the end of the paper considerably here. We have added in a distinct conclusions section so that we can more clearly

consideration.	articulate the outcomes of the research to the reader.<br>We have retained the results and discussion as a combined section as the complexity of the results called for the inclusion of the discussion at the same time so that we could explain each part of the interpretation in context. We have however revised much of this to improve the clarity and flow for the reader as well.
**JOURNAL REQUIREMENTS**	
**Comment**	**Reply**
1. Please ensure that your manuscript meets PLOS ONE's style requirements, including those for file naming. The PLOS ONE style templates can be found at<br>https://journals.plos.org/plosone/s/file?id=wjVg/PLOSOne_formatting_sample_main_body.pdf and<br>https://journals.plos.org/plosone/s/file?id=ba62/PLOSOne_formatting_sample_title_authors_affiliations.pdf	We have checked formatting.
2. Please provide additional details regarding participant consent. In the ethics statement in the Methods and online submission information, please ensure that you have specified (1) whether consent was informed and (2) what type you obtained (for instance, written or verbal, and if verbal, how it was documented and witnessed). If your study included minors, state whether you obtained consent from parents or guardians. If the need for consent was waived by the ethics committee, please include this information.<br>If you are reporting a retrospective study of medical records or archived samples, please ensure that you have discussed whether all data were fully anonymized before you accessed them and/or whether the IRB or ethics committee waived the requirement for informed consent. If patients provided informed written consent to have data from their medical records used in research, please include this information.	The details of the provision of an explanatory statement and that consent was obtained through a button press on the computer is now included in the main manuscript.
3. Thank you for stating the following in the Acknowledgments Section of your manuscript:<br>"We thank Harmen Oppewal and the Monash Business Behavioral Laboratory for facilities and support; Funding: A.G.D. received funding support from the Australian Research Council (LE130100112), S.R.H. was supported by an Alfred Deakin Research Fellowship"	We have now removed that funding information from the Acknowledgements and confirm that the current funding statement you describe is suitable.

Please note that funding information should not appear in the Acknowledgments section or other areas of your manuscript. We will only publish funding information present in the Funding Statement section of the online submission form.<br>Please remove any funding-related text from the manuscript and let us know how you would like to update your Funding Statement. Currently, your Funding Statement reads as follows:<br>"A.G.D. received funding support from the Australian Research Council (LE130100112), S.R.H. was supported by an Alfred Deakin Research Fellowship."<br>Please include your amended statements within your cover letter; we will change the online submission form on your behalf.	
4. Thank you for stating the following financial disclosure:<br>"A.G.D. received funding support from the Australian Research Council (LE130100112), S.R.H. was supported by an Alfred Deakin Research Fellowship."<br>Please state what role the funders took in the study. If the funders had no role, please state: "The funders had no role in study design, data collection and analysis, decision to publish, or preparation of the manuscript."<br>If this statement is not correct you must amend it as needed.<br>Please include this amended Role of Funder statement in your cover letter; we will change the online submission form on your behalf.	We can confirm that "The funders had no role in study design, data collection and analysis, decision to publish, or preparation of the manuscript" and are happy for that to be included in the statement.
Please review your reference list to ensure that it is complete and correct. If you have cited papers that have been retracted, please include the rationale for doing so in the manuscript text, or remove these references and replace them with relevant current references. Any changes to the reference list should be mentioned in the rebuttal letter that accompanies your revised manuscript. If you need to cite a retracted article, indicate the article's retracted status in the References list and also include a citation and full reference for the retraction notice.	We have complied with this and added references are as per reviewer requests detailed in these responses.
**REVIEWER ONE**	
**Comment**	**Reply**
The aim of the present study was to investigate whether there is a dominant dimension (horizontal vs vertical) in Spatial Numerical Association of Response Codes (SNARC). The task was a	We have made some substantial revisions to the paper, one of the most important being to more adequately address the native language component of the analysis. We have provided a better integration of the

comparison task where pairs of numbers are placed along the diagonal axes and the position of the largest number (the target) must be identified. The results show that participants respond more efficiently in vertical than horizonal orientations when both numbers being compared are beyond the range of 1-4; furthermore, when the target is in the upward position the response time decreasing significatively, but only in a subset of data including responses from participants whose language was not exclusively expressed in a left to right orientation (E.g. Cantonese and Korean). I think that the manuscript has several methodological issues.	results, namely that the correct choices data supports the vertical dominance, and the response time data for the LTR native language group (the larger of the two groups for this moderator and the group that matches the language of the experiment) also support the vertical dominance. We now better explain the role of language as a potential boundary condition/moderator to the results presented, and emphasize that it was derived from post-hoc analysis. Indeed, consistent with our granted ethics, we cannot pre-screen for such language (cultural) factors, and this factor was only a result of information that volunteer participants elected to supply.
The type of task used is interesting and could add to the literature concerning this line of research. However, the sample is unbalanced as it includes 63% of participants whose native language exclusively uses a left-to-right (LTR) oriented script and 37% of participants whose native languages employed a mixture of script orientations which may be presented LTR or Top to Bottom. The authors should have used a sample composed only of participants who exclusively use a LTR; alternatively, if the authors wanted to compare different reading-writing directions groups, the sample had to be balanced 50% / 50%. Their results and conclusions are affected by their sampling.	We have revised the paper considerably to better address the role of native language orientation in the paper.<br><br>Language was not a hypothesis proposed when the data was collected. The potential dominance of one orientation of the SNARC was the research hypothesis. Native language was only introduced because of the incidental detection within the demographic variables that the non-LTR group composed 37% of the sample. We did not want to balance on native language, we sought only to recruit fluent English speakers.<br><br>We have revised the presentation of results including the demographic variables (including native language) and show how our results make an important contribution by addressing the dominant orientation of SNA effects, and the potential moderating role of native language in SNAs.<br><br>We would also caution that the LTR and non-LTR native language groups should be treated with caution, as they are an artificial grouping of languages for the convenient treatment of a covariate in our analysis. 'non-LTR' conceptually could include left to right, top to bottom, bottom to top language orientations. So the three non-LTR and single LTR orientations would imply FOUR groups, and some languages, such as Mandarin can be flexibly written TTB and/or LTR implying even more. Our grouping was made only to address the incidental inclusion of a reasonable sized group of non-LTR native language speakers. Under a hypothesis of native language playing a role then substantial more work on selecting native language groups would be required. Our research does not have the ethics for this as there is an obvious implication of culture and race that does not form a part of our research question.<br><br>We do note that our discovery with this effect does motivate future experimental work involving native language though. Now that there is

	some data to support a hypothesis of the impact of native language (and not just spoken language in general) on SNARC there is a basis for an ethics application to examine this research. Future research has the potential to unpack this incidental discovery of our further; but that was not our research goal in this study.
Secondarily, in its present form, the study seems "incomplete" as participants were always asked to identify only the largest number of the pair (the target); to be in line with SNARC effect, the authors should also have included in the study responses when the target was the smallest number.	This is an important point highlighted by the editor as well that we attend to carefully. We have provided a full explanation of our reasoning and theoretical basis for our choices in our response above to the editors' comment.
In general, the article not well-structured (for example the hypotheses of the study are not defined) and contains, in addition to the aforementioned methodological problems, many parts to be rewritten and better explained. Please find below some comments concerning other critical aspects (major points only).	We have revised the paper and now include a specific research hypothesis and have re-organized the method and results to better guide the reader through the research. We have also now included a conclusions section so that the meaning of the results can be better understood by the reader.
Abstract<br>- P3, line 49-51 : What does the task consist of? the description is not clear; please specify	A more elaborate description of the task has now been supplied in the abstract.
- P3, line 51-53: "Results show that humans are faster at number comparisons in vertical rather than horizontal orientations, but only when both numbers being compared are beyond the range of 1-4 and the largest number is in the upward position". The results indeed shows that the participants when both numbers being compared are beyond the range of 1-4 respond more efficiently (the proportion of correct choices is higher) but not faster. Furthermore, the faster response when the target was in the upward position was found only with a subset of participants. Please correct the inaccuracy.	We have completely re-written the results component of the abstract here to focus on the main result that was the basis for the research hypothesis, and better framed the native language moderating effect.
- P4, line 65-69: The SNARC effect is not alone the association between numbers and space along a mental number-line oriented from left to right with small number on the left (e.g. 1,2) and large numbers on the right (eg. 8,9); in fact, it refers also to the phenomenon that individuals typically react faster to relatively smaller numbers with left-sided responses and faster to relatively larger numbers with right-sided responses. The authors should add this description	Due to limited space in the abstract we have refocused it away from the numerical distance and magnitude effects, which were not the main focus of the experiment (not hypothesized). These results still get a full discussion in the paper though.
Introduction<br>- P6, line 135: Which is the "innate biases across dimension"?	This phrase has been removed.

Method<br>- P8, line 162-164: How the angle of the joystick response was translated into perceived dominant preference for horizontal (left / right) or vertical (up /down) orientation?	We have added a statement explaining that this refers to us making the 'corner' being responded to being interpreted as a function of the combined horizontal and vertical movements involved.
- P8, line 185: I suggest replacing the word "people" with participants.	This section has now been completely re-written in response to other comments.
Results and Discussion<br>- P9, line 201: The authors should divide the results and discussion; furthermore, the manuscript does not present a proper discussion.	Due to the complexity of the results in the analysis we have opted to retain the results and discussion in the same section. We have however included a conclusions section so that the substantive outcome of the research can be better articulated.
- P11, line 239-242: the meaning of this section in unclear; it should be rewritten more clearly.	This section of the results have been completely revised to improve clarity.
- P12, line 268: "special-numerical associations" makes little sense; please, replace with "spatial-numerical associations".	This has been corrected.
- P12, line 274-275: "Results show that numbers are responded to more efficiently when a larger number is in the upwards vertical position with the horizontal position having no impact on response efficiency, suggesting that the dominant mental representation of number is vertical not horizontal". It is wrong to say that in the numerical representation the dominant dimension is the vertical as the results were found only in a subset of participants (people whose native languages employed Left to Right or Top To Bottom script orientations) furthermore, the significant effect in this subset of participants could be due to the native language of this participants.	We have completely revised the parts of the analysis and the write up of this in response to the issue you highlighted here. We have no more clearly presented both language groups and discuss the moderating role of native language for the efficiency/reaction time data. We also highlight that the accuracy/correct choice supports the vertical conclusion, along with the LTR subset for efficiency.
**REVIEWER TWO**	
**Comment**	**Reply**
Dear Authors, I am pleased to review a manuscript that I consider informative in the field of spatial-numerical associations (SNAs). I recommend this article for publication as it brings a valid methodological and theoretical contribution but only after making major modifications and clarifications raised by the comments below. I list them following the order of the article's main sections.	Thank you for your insight into the paper here. We have responded to each of your comments below.
ABSTRACT<br>In the Abstract, the number comparison task is not defined (e.g., the task required participants to indicate which of two, visually displayed, single-digit numbers was larger).	We have revised this part of the abstract in particular so that the task is much more apparent to the reader.
INTRODUCTION:	We have updated our use of terminology here to be more specific and

1. In order to better contextualize the authors' contribution in the field of SNAs, I would suggest them to use the recent terminology coined by Cipora and colleagues (2020; doi: 10.3389/fpsyg.2020.00182) and by Shaki and Fischer (2018; doi.org/10.1016/j.cognition.2018.02.022). Indeed, with the authors' novel method described in this study, they aim to assess "Direction SNAs" during "explicit magnitude processing" and "explicit spatial directional processing"	integrated this work into the paper. This has also helped frame our contribution more specifically.
2. In order to give a more comprehensive overview of the literature, I recommend the authors to consider the following two articles:<br>- Sixtus, E., Lonnemann, J., Fischer, M. H., & Werner, K. (2019). Mental number representations in 2D space. Frontiers in psychology, 10, 172. https://doi.org/10.3389/fpsyg.2019.00172. This study assessed "Direction SNAs" during "implicit magnitude processing" and "implicit spatial directional processing" along the horizontal and vertical axes;<br>- Lohmann, J., Schroeder, P. A., Nuerk, H. C., Plewnia, C., & Butz, M. V. (2018). How deep is your SNARC? Interactions between numerical magnitude, response hands, and reachability in peripersonal space. Frontiers in psychology, 9, 622. https://doi.org/10.3389/fpsyg.2018.00622. This study assessed interactions between horizontal and radial spatial-numerical mappings in a virtual reality environment;<br>- Felisatti, A., Ranzini, M., Blini, E., Lisi, M., & Zorzi, M. (2022). Effects of attentional shifts along the vertical axis on number processing: An eye-tracking study with optokinetic stimulation. Cognition, 221, 104991. https://doi.org/10.1016/j.cognition.2021.104991. This study revealed bi-directional links between numbers and attentional orienting along the vertical axis.	These have now been integrated. The first has been put into the conclusions as it fits into a different paradigm than the one used in our research.
3. I recommend the authors to explicitly describe the hypotheses.	We have now formally introduced the research hypothesis for this study.
METHOD:<br>1. Even if spaces are used to separate different subsections, I would suggest the authors to use also different subtitles. These would enable the reader to better organize the methodological and conceptual parts.	We have re-arranged the method and added in subtitles to assist the reader in navigating the information here.
2. Participants: Did the authors record information about the	Yes this was recorded. A demographics section has now been added to

handedness of the participants and their familiarity with joystick-related activities and games?	results providing this information for the reader.
3. Cultural influence: Evidence has shown that not only reading direction habits but also counting direction habits play a role in spatial-numerical associations. I suggest the authors to consider this aspect (Shaki, S., Fischer, M. H., & Petrusic, W. M. (2009). Reading habits for both words and numbers contribute to the SNARC effect. Psychonomic bulletin & review, 16(2), 328-331. https://doi.org/10.3758/PBR.16.2.328)	We have made a more explicit mention of habituation in our conclusions section, as it may play an important part in the language covariate. The number direction too may also play a role and we have incorporated that insight, but cannot test that covariate in our analysis as we do not have that data available to us.
4. Material: A picture of the setting would be useful to visualize the participant-joystick interaction	We did not control the participant-joystick interaction as we wanted participants to select the most natural grip type for themselves. We have now explicitly described that in the text. We have also expanded the description for the image of the experimental setting so that this and other parts of the experimental setup are clearer for the reader.<br><br>Anecdotally, we did see at least two grip types in use by participants. One had the palm over the top of the ball clasping in a downwards manner. The other had the palm more on the side of the ball clasping in a sideways manner. We have not recorded this in the manuscript though as we have no systematic observations of grip type, and there are likely many more than the two we describe here.
5. Sequence of event: A picture showing the timeline and the sequence of event would be informative	We have done two things in response to this request. We have added a section in the method 'Experimental Task' where the sequence of events is now made explicit. We have also added additional explanation to the current image of the experimental setting.
6. Task confound: An additional block (counterbalanced within or even between participants) asking participants to indicate the smaller and not the larger between the two numbers would have been very informative. Indeed, the unique association between large numbers with upward space might also be related to focus only on the relative larger numbers	This is an important issue you raise that the editor particularly drew our attention to. We have provided a full discussion for this in our response to the editor above.
7. Spatial confound: Since the main aim of the study was to control for methodological confounds related to motor response, I would have expected a method without any spatial connotations. Instead, in the study both the stimulus encoding and the response were lateralized. Moreover, the device entailed confounds between the radial and the vertical dimensions. I suggest the authors to report more in detail the reasons motivating each methodological decision.	We had intended to use an explicitly spatial task, which necessarily draw in a spatial response format. We have better framed the research though so that this choice is much clearer to the reader. We have also reframed some of our review of the present literature to focus more on the bilateral response issues typical of explicit spatial tasks.<br>We did necessarily confound radial joystick motion with vertical spatial orientation. We have now explained this choice, which was done

	because the need to resist gravity by participants would likely induce effects and fatigue.
RESULTS AND DISCUSSION<br>1. I suggest the authors to subdivide the different sections by adding subtitles (e.g., Accuracy preprocessing and results, Reaction time preprocessing and results, Discussion)	We have rearranged the results here so that there is a clearer flow overall. We have added an extra demographics section that sets up some of the subsequent analysis more clearly and have created a separate conclusions section to improve clarity. We could not divide up the result too much as we found it started to disrupt the flow, but how our changes resolve the concerns raised here.
2. The Discussion part is not comprehensive. I recommend the authors to further elaborate it by interpreting the results in light of the literature considered in the Introduction. Moreover, I would suggest them to add a “Limitation and future directions” section where to report the confounds/limitations of their method and their solutions to overcome them in future research.	The editor also noted the need to provide some restructuring at the end of the paper. We have provided a more full response there in light of their and your comments.
