# Supplementary material for: Vertical versus horizontal Spatial-Numerical Associations (SNA): A processing advantage for the vertical dimension
Source: PLoS One. 2022 Aug 25;17(8):e0262559. doi: 10.1371/journal.pone.0262559 (PMC9409557; doi:10.1371/journal.pone.0262559)
Supplement: Attachment — Submitted filename: PLOS Response to reviews_plain_agd.docx [file pone.0262559.s002.docx]

| **COMMENT** | **RESPONSE** |
| --- | --- |
| EDITOR | |
| Thank you for submitting your manuscript to PLOS ONE. I have sent it to a reviewer of the  previous version. As you can see below, the reviewer thinks that the manuscript is much improved. I also think that you addressed the previous comments satisfactorily and am happy to accept the manuscript for publication in PLOS ONE, pending the minor modifications  recommended by the reviewer. | Thank you, we have made requested final revisions as requested and detailed below.  Please note that during review process corresponding author Adrian G. Dyer changed universities, which was logged at the time via email to PLoS (and in system), and thus reflected in address change on revised manuscript. |
| REVIEWER 1 | |
| Dear Editor and Authors,  I am happy to endorse the publication of the article "Vertical versus Horizontal Spatial  Numerical Associations (SNAs): A processing advantage for the vertical dimension". The  Authors addressed all the points raised by me in a comprehensive way, adding conceptual and  methodological clarifications. However, I would recommend the Authors to make few more  modifications: | Thank you |
| 1) HYPOTHESIS: Specify that they predict prevalence of one dimension and motivate it, for  instance by taking into account the Hierarchical view (Fischer, 2012), according to which the  vertical dimension is considered the most grounded and universal one; | For our hypothesis we didn’t seek to hypothesize that the vertical dimension would be dominant. Our hypothesis was simply that one dimension would be dominant (the null being no dominant dimension). We agree that Fischer (2012) would suggest the vertical is more grounded (which is a view in line with our own intuition), but there have been empirical results that suggest alternatives, such as Holmes (2012) finding the horizontal is dominant. While we believe these alternative studies were impacted by response bias, something we highlight in our paper, we didn’t want to completely dismiss their findings at the hypothesis stage of our paper. We sought to control for the bias and then see what the results of our experiment showed. In this case, our results find the vertical dominates in line with Fischer (2012). We believe this approach to writing the hypothesis presents a more ‘neutral’ review of the literature in line with how we sought to balance the current apparent contradictions in literature. |
| 2) METHOD: Explicitly report in the text the reasons why they did not include the block  involving response to the smaller number. I suspect that many readers would have the same  question; | We have now included this discussion in the method. |
| 3) DISCUSSION: Insert a paragraph describing the limitations of the study. | We have added a discussion of the limitations particularly focusing on our apparatus in this case. While the apparatus overcome several biases present in other studies it presents some new challenges for some paradigms. These are introduced and discussed. |
